# Loneliness and health risk behaviours among Russian and U.S. adolescents: a cross-sectional study

**DOI:** 10.1186/1471-2458-14-366

**Published:** 2014-04-16

**Authors:** Andrew Stickley, Ai Koyanagi, Roman Koposov, Mary Schwab-Stone, Vladislav Ruchkin

**Affiliations:** 1 Stockholm Centre on Health of Societies in Transition (SCOHOST), Södertörn University, 141 89 Huddinge, Sweden; 2 Department of Human Ecology, Graduate School of Medicine, University of Tokyo, Tokyo, Japan; 3 Regional Centre for Child and Youth Mental Health and Child Welfare, UiT The Arctic University of Norway, Tromsø, Norway; 4 Child Study Centre, Yale University Medical School, New Haven, CT, USA; 5 Department of Social and Forensic Psychiatry, Division of Neuroscience, Karolinska Institute, Huddinge, Sweden

**Keywords:** Adolescent, Health risk behaviour, Loneliness, Russia, United States

## Abstract

**Background:**

For some adolescents feeling lonely can be a protracted and painful experience. It has been suggested that engaging in health risk behaviours such as substance use and sexual behaviour may be a way of coping with the distress arising from loneliness during adolescence. However, the association between loneliness and health risk behaviour has been little studied to date. To address this research gap, the current study examined this relation among Russian and U.S. adolescents.

**Methods:**

Data were used from the Social and Health Assessment (SAHA), a school-based survey conducted in 2003. A total of 1995 Russian and 2050 U.S. students aged 13–15 years old were included in the analysis. Logistic regression was used to examine the association between loneliness and substance use, sexual risk behaviour, and violence.

**Results:**

After adjusting for demographic characteristics and depressive symptoms, loneliness was associated with a significantly increased risk of adolescent substance use in both Russia and the United States. Lonely Russian girls were significantly more likely to have used marijuana (odds ratio [OR]: 2.28; confidence interval [CI]: 1.17–4.45), while lonely Russian boys had higher odds for past 30-day smoking (OR, 1.87; CI, 1.08–3.24). In the U.S. loneliness was associated with the lifetime use of illicit drugs (excepting marijuana) among boys (OR, 3.09; CI, 1.41–6.77) and with lifetime marijuana use (OR, 1.79; CI, 1.26–2.55), past 30-day alcohol consumption (OR, 1.80; CI, 1.18–2.75) and past 30-day binge drinking (OR, 2.40; CI, 1.56–3.70) among girls. The only relation between loneliness and sexual risk behaviour was among Russian girls, where loneliness was associated with significantly higher odds for ever having been pregnant (OR, 1.69; CI: 1.12–2.54). Loneliness was not associated with violent behaviour among boys or girls in either country.

**Conclusion:**

Loneliness is associated with adolescent health risk behaviour among boys and girls in both Russia and the United States. Further research is now needed in both settings using quantitative and qualitative methods to better understand the association between loneliness and health risk behaviours so that effective interventions can be designed and implemented to mitigate loneliness and its effects on adolescent well-being.

## Background

Loneliness is commonplace during adolescence [1,2]. One recent review reported that up to 80% of those aged under 18 feel lonely at least sometimes [3]. This high prevalence of loneliness may emanate from the multitude of developmental and social changes that take place during the adolescent years. Physical and cognitive growth, the disruption of the pre-adolescent self concept, and the need for greater individuation and autonomy may all make young people especially vulnerable to loneliness [4]. More specifically, a wide range of circumstances that can include personal characteristics such as shyness, low self-esteem and poor social skills [4], mistaken expectations about new social relations [1], and increasing conflict with parents [5], all increase the likelihood of loneliness, that is, the unpleasant experience that arises from perceiving one’s social relations as being inadequate in terms of satisfying important social needs [3,6].

Loneliness can be an extremely painful and distressing phenomenon [7] that can have serious consequences for adolescent well-being. In the educational sphere, it has been linked to increased truancy [6], worse academic performance [8] and a greater likelihood of dropping out of school [1]. In terms of health outcomes, loneliness has been related to worse health perceptions [9], somatic symptoms such as headaches [10], poorer psychological health including anxiety [11] and depression [12], as well as to an increased risk for suicidal behaviour [13].

One way adolescents might respond to loneliness is by pursuing ‘alternative gratifications’ in order to cope with, or minimise, the painful feelings that can emanate from loneliness [4]. These might include risky health behaviours. Several studies have shown for example, that lonely adolescents are more likely to use alcohol, cigarettes and illicit drugs [14-16], possibly as a form of self-medication in response to the pain of loneliness, although psychological distress might also make adolescents more vulnerable to peer influence concerning the use of substances [16]. Emotions might also influence adolescent sexual behaviour [17], as loneliness has been linked to the early initiation of sexual activity among adolescents [18], as well as to sexual risk behaviours such as less consistent condom use [19]. These associations may be underpinned by the belief that engaging in sex is one way to reduce feelings of loneliness [20], with intimate connections possibly being seen as a way to counter social rejection [21]. Feeling lonely may also be connected with aggressive behaviour [22-24]. Specifically, being aggressive with peers may lead to worse relations and possibly subsequent rejection [23,24], although loneliness in childhood has also been found to be a precursor of later aggression in adolescence [22]. As loneliness has been linked to peer victimisation [25], it is possible that victimised and lonely adolescents may be engaging in aggressive and violent behaviours (such as weapon carrying) out of fear and in the desire to protect themselves [26].

Despite these studies, until now, there has been little systematic research on the effect of loneliness on adolescent health behaviour. The few studies that focus exclusively on this topic have been concentrated in North America. Even within this body of research there has been a tendency to concentrate on substance use and physical activity [14,15,27]. This is an important research gap as loneliness and health risk behaviours can vary between countries, and also between boys and girls within countries [16,28,29]. A recent study which examined loneliness and substance use among adolescents in four countries found for example, that in every country (Chile, China, Namibia and the Philippines) lonely adolescents were significantly more likely to be current drinkers [16]. However, among Seychelles’ adolescents loneliness was not linked to alcohol use [28]. Similarly, while lonely boys and girls were more likely to smoke in Chile and Namibia, Filipino boys and Chinese girls who were lonely did not have elevated odds for smoking, even though their country opposite sex counterparts did [16]. These contrasting results suggest that cross-country research may be important when it comes to understanding the relation between adolescent loneliness and health risk behaviour.

The current study will thus examine loneliness and its association with various adolescent health risk behaviours among boys and girls in two countries that are historically and culturally distinct: Russia and the United States. This study will extend research on the effects of loneliness firstly, by focusing on its relation with adolescent health risk behaviours (i.e. sexual and violent behaviour) that have been little researched to date. Secondly, it will extend this research to Eastern Europe where there has been little research on loneliness in general. Indeed, Russia may provide an ideal location to examine the relation between adolescent loneliness and health risk behaviour as the prevalence of loneliness among the population has been reported to be comparatively high there [30]. Furthermore, recent studies have shown that loneliness might not only be impacting on population health in Russia [31], but that it might also be linked to adult [31] and adolescent [32] health risk behaviours in varying ways in the former Soviet countries. The following hypotheses will be examined: (1) That lonely adolescents are at an increased risk of engaging in health risk behaviours; (2) That the relationship between adolescent loneliness and health risk behaviour can vary by the type of health risk behaviour.

## Methods

### Study participants

Data in the present study were drawn from surveys undertaken in Russia and the U.S. in spring 2003 as part of the Social and Health Assessment (SAHA) study using a common survey instrument in both countries [33]. Surveys were undertaken in the northern Russian city of Arkhangelsk and in the city of New Haven in the north-eastern United States (Connecticut). In Arkhangelsk, data were collected from a representative sample of students in the sixth to tenth grades (age 12–17) in the city’s public schools (i.e. 10% of students in each of the city’s four districts), while in New Haven, all students aged 13–17 who were in the urban public school system were recruited. In Russia, subjects were selected from schools that were randomly selected from a list of all schools in each of the city’s districts, and from within classes randomly selected from within each school. All of the students within each class were invited to participate in the survey. Students in both countries completed the survey questionnaire themselves in their classrooms in the presence of their teachers during a normal school day. All information was collected anonymously (that is, there was no means of identifying completed individual student questionnaires). Written informed consent was given by all participants before the questionnaire was administered and both parents (on behalf of their children) and children had the right to refuse to participate in the study. In Russia, the recruited sample comprised 2892 students (refusal rate 3.6%) while in the U.S. the sample size was 2631 (refusal rate <1%).

### Ethical approval

Ethical approval for the study was obtained from the Northern State Medical University in Arkhangelsk, Russia and Yale University School of Medicine in the United States.

### Measures

Items from the SAHA survey questionnaire were used in the current study [33]. The survey included new scales that were developed specifically for the SAHA study and scales that had been used previously with similar populations. In the current study, the following measures were used:

#### Loneliness

Following the lead of a recent study [34], in the present work, the loneliness question was drawn from an adapted version of the Centre for Epidemiological Studies-Depression Scale (CES-D) [35]. Students were asked to think about how they ‘felt or behaved in the past 30 days’. In response to the statement ‘I felt lonely’, students were presented with the response options ‘Not true’, ‘Somewhat true’ and ‘Certainly true’. As loneliness can be a transitory feeling that can be experienced by almost anyone from time-to-time, in this study, we focused on those expressing the most intense feelings of loneliness. Hence, ‘Not true’ and ‘Somewhat true’ were used as the reference category (No) while ‘Certainly true’ was taken as signifying feeling lonely (Yes).

#### Substance use

Information was gathered on five forms of substance use. Smoking behaviour was assessed with the question, ‘During the past 30 days, on how many days did you smoke?’ The answer was dichotomised to 0 days and on one or more days. Lifetime marijuana use was assessed with the question, ‘Have you ever used marijuana?’ Respondents were categorised as either never-users or users. Information was also obtained about lifetime use of other illicit drugs – glue/aerosols; amphetamines, ecstasy or dust; and LSD (lysergic acid diethylamide [acid])/PCP (phencyclidine [angel dust]). Students were then dichotomised into users (those who answered yes to ever using any of these substances) and non-users. Two measures were also examined in relation to drinking alcohol. Adolescents who answered that they had consumed either beer, wine or hard liquor at least once in the past 30 days were categorised as current alcohol users. The second alcohol measure related to binge drinking and was assessed by responses to the question, ‘During the past 30 days, on how many days, if any, did you have five or more drinks of alcohol in a row, that is, within a couple of hours?’ Responses were dichotomised to represent those who had engaged in binge drinking on one or more days and those who did not binge drink on any days.

#### Sexual behaviour

Four questions were asked about students’ sexual behaviour. Information on lifetime sex was obtained by asking ‘How many people have you had sexual intercourse with (“gone all the way”)?’ Answers were dichotomised into had sexual intercourse and never had sexual intercourse. Details of substance use during the students’ last sexual encounter were obtained by asking, ‘The last time you had sexual intercourse, had you been drinking alcohol or using drugs?’ Answer options were ‘I’ve never had sexual intercourse’, ‘Yes’, and ‘No’. The analysis was restricted to those who had previously engaged in sex. Using the same answer options and categorisation scheme just mentioned, information on condom use at last sex was obtained by asking, ‘The last time you had sex did you or your partner use a condom?’ Students were also asked ‘How many times have you been pregnant or got someone pregnant?’ with response options 0 times (No) vs. 1 time or 2 or more times (Yes).

#### Violent behaviour

Three questions related to the students’ experience of violence in the past year. They were asked how many times (if any) they had ‘started a fistfight or shoving match’, ‘hurt someone badly in a physical fight so that they had to be treated by a doctor or nurse’ or ‘carried a blade, knife, or gun in school’. For the present study these answers were dichotomised into 0 times and 1 or more times.

#### Control variables

Information on the age of the students and on the educational level of their parents/guardians was also used. This latter variable has been linked to both differences in levels of reported adolescent loneliness [36] and adolescent health risk behaviours such as getting drunk [37] and smoking [38]. In the present study it was dichotomised into graduated from college (high education) and having less than a college graduate’s education (low education). If both parents (or the male or female guardian) were present in the home, the highest educational level was used as the category for that family. Students were also categorised as living in family structures where both biological parents were present (i.e. their family was intact), or as living with a single parent where there was one biological parent and no step parent. Family living arrangements that differed from either of the aforementioned forms were categorised as ‘other’. We controlled for family structure as some previous research has suggested that adolescents not living with both biological parents report more loneliness [39] and are more likely to engage in violence, use substances such as tobacco and alcohol as well as have sex [40].

Finally, as research has indicated that there may be a close relation between loneliness and depression [41], and that depression may itself be a risk factor for poorer adolescent health behaviour [42], we also controlled for the effects of depressive symptoms in the regression analysis (which was not done in those earlier studies that focused specifically on the relationship between adolescent loneliness and health risk behaviours [14,15,27]). Depressive symptoms were assessed by the use of a composite score based on the CES-D after removing the question on loneliness - a method that has been used in a previous study [43]. Those within the highest quintile of scores were classified as having depressive symptoms. There was a high degree of internal consistency for this measure among both Russian (Cronbach’s α = 0.79) and U.S. (Cronbach’s α = 0.88) students.

### Statistical analysis

For comparability between the countries, the analysis was restricted to those aged 13-15 years old. After excluding those out of this age range and the 102 students with no information on sex and/or age, the final sample size was 1995 in Russia (1105 girls and 890 boys) and 2050 in the United States (1024 girls and 1026 boys). Differences in the prevalence of loneliness were examined with chi-square tests. Univariate and multivariate logistic regression analyses were carried out to assess the association between loneliness and the health risk behaviours. Analyses were stratified by sex because as mentioned previously, there is some evidence that the effects of loneliness on health risk behaviour may differ between boys and girls [14]. Three models were constructed. In Model 1, the univariate relation between loneliness and each individual health risk behaviour was examined. In Model 2, these relations were examined while controlling for age, parental education, and family structure. Model 3 was exactly the same as Model 2 except for the addition of the depressive symptoms variable. These control variables were all categorical variables. Due to the large number of missing cases for parental education (22% for Russia and 17% for the United States), a missing category (coded 2) was included in the regression analysis together with low and high education coded as 0 and 1 respectively. The analyses on last sex alcohol/drug use and last sex non-condom use were restricted to those who had ever had sex. The clustering effect within schools was adjusted for in all analyses by the use of a sandwich estimator. Results are presented in the form of odds ratios (OR) with 95% confidence intervals (CI). The analyses were performed using the statistical software package Stata 12.1 (Stata Corp LP, College Station, Texas) with p < 0.05 signifying statistical significance.

## Results

The characteristics of the study sample are presented in Table 1. The U.S. sample was younger with fewer intact families, and a lower level of parental education. In terms of the prevalence of loneliness, there was little difference between Russian and U.S. girls (14.4% vs. 14.7%; p = 0.844), while the prevalence of feeling lonely was slightly higher for Russian boys than their U.S. counterparts (8.9% vs. 6.7%; p = 0.080).

**Table 1:** Characteristics of the study sample

		**Russia**				**U.S.**			
		**Female**		**Male**		**Female**		**Male**	
**Characteristic**	**Categories**	**n**	**%**^**†**^	**n**	**%**^**†**^	**n**	**%**^**†**^	**n**	**%**^**†**^
**Demographic characteristics**									
Age (years)	13	150	13.6	127	14.3	611	59.7	550	53.6
	14	462	41.8	399	44.8	357	34.9	381	37.1
	15	493	44.6	364	40.9	56	5.5	95	9.3
Parental education	Low	330	38.0	278	40.4	553	64.8	532	62.4
	High	538	62.0	410	59.6	301	35.2	320	37.6
Family structure	Intact	730	66.4	571	64.3	320	31.3	371	36.2
	Single	266	24.2	221	24.9	405	39.6	373	36.4
	Other	104	9.5	96	10.8	299	29.2	282	27.5
**Substance use**									
Smoking (past 30 days)		315	31.1	296	37.1	106	11.2	64	7.0
Lifetime marijuana use		66	6.2	64	8.1	161	16.6	181	19.3
Lifetime other illicit drug use^§^		44	4.1	73	8.7	65	6.6	68	7.1
Alcohol consumption^¶^ (past 30 days)		689	64.3	513	61.7	271	27.9	273	29.3
Binge drinking^#^ (past 30 days)		396	37.3	280	34.7	123	12.8	105	11.4
**Sexual behaviour**									
Lifetime sex		116	11.4	176	23.0	211	22.5	412	48.1
Last sex alcohol or drug use^ǂ^	No	106	82.2	134	68.0	211	92.5	421	92.3
	Yes	23	17.8	63	32.0	17	7.5	35	7.7
Last sex condom use^ǂ^	No	92	71.9	64	32.8	60	26.8	92	20.6
	Yes	36	28.1	131	67.2	164	73.2	355	79.4
Got someone pregnant/been pregnant		51	4.9	42	5.2	26	2.7	52	5.7
**Violence** (past year)									
Started a fistfight or shoving match		215	20.0	384	45.9	319	32.5	425	44.8
Hurt someone badly in a physical fight		60	5.6	139	16.8	171	17.5	243	25.6
Carried a blade, knife, or gun in school		56	5.2	134	16.1	100	10.3	166	17.7
**Personal characteristic** (past 30 days)									
I felt lonely (certainly true)		153	14.4	75	8.9	146	14.7	64	6.7

^†^Percentages are calculated based on the sample with no missing values for that variable. Percentages for last sex alcohol or drug use and last sex condom use are based on those who ever had sex.

^§^Illicit drug use includes huffing to get high (glue, aerosols), amphetamines, ecstasy or dust, LSD (Acid) or PCP.

^¶^Alcohol consumption was based on the consumption of either beer, wine or hard liquor.

^#^Binge drinking referred to drinking at least five drinks of alcohol in a row on at least one day.

^ǂ^Restricted to those who ever had sex.

In the univariate analysis, lonely Russian girls had higher odds for smoking, marijuana use, binge drinking, lifetime sex, and having been pregnant (Table 2). Controlling for the effects of the demographic variables in Model 2 made little difference to these associations (or more generally to the association between loneliness and any of the health risk behaviours for girls and boys in either country). The addition of the depressive symptoms variable in Model 3 however, attenuated the strength of many of these relations so that only two remained statistically significant: lonely Russian girls were more likely to have smoked marijuana (odds ratio [OR], 2.28; confidence interval [CI], 1.17–4.45) and been pregnant, (OR, 1.69; CI, 1.12–2.54). Among Russian boys, in the univariate analysis, feeling lonely was associated with higher odds for smoking, lifetime marijuana use, other illicit drug use, last sex substance use, getting someone pregnant and weapon carrying in school (Table 2). After controlling for depressive symptoms in Model 3 the only association which remained statistically significant was with smoking, with lonely Russian boys having higher odds for smoking compared with their non-lonely counterparts (OR, 1.87; CI, 1.08–3.24).

**Table 2:** Univariate and multivariate analyses on the association between loneliness and health risk behaviours among Russian adolescents

		**Girls**			**Boys**		
		**Model 1**	**Model 2**	**Model 3**	**Model 1**	**Model 2**	**Model 3**
**Health risk behaviours (outcome)**	**I felt lonely**^**¤**^	**OR (95% CI)**	**adj. OR (95% CI)**^**†**^	**adj. OR (95% CI)**^**ǂ**^	**OR (95% CI)**	**adj. OR (95% CI)**^**†**^	**adj. OR (95% CI)**^**ǂ**^
**Substance use**							
Smoking (past 30 days)	No	1.00	1.00	1.00	1.00	1.00	1.00
	Yes	1.62 (1.20–2.19)^b^	1.52 (1.10–2.09)^a^	1.10 (0.79–1.52)	2.17 (1.42–3.32)^c^	2.13 (1.42–3.20)^c^	1.87 (1.08–3.24)^a^
Lifetime marijuana use	No	1.00	1.00	1.00	1.00	1.00	1.00
	Yes	2.22 (1.11–4.44)^a^	2.06 (1.00–4.25)	2.28 (1.17–4.45)^a^	1.84 (1.07–3.17)^a^	1.81 (1.28–2.56)^b^	1.49 (0.74–3.00)
Lifetime other illicit drug use^§^	No	1.00	1.00	1.00	1.00	1.00	1.00
	Yes	1.35 (0.87–2.11)	1.37 (0.87–2.15)	0.90 (0.62–1.30)	2.74 (1.56–4.81)^c^	2.83 (1.55–5.17)^b^	1.59 (0.85–2.96)
Alcohol consumption^¶^ (past 30 days)	No	1.00	1.00	1.00	1.00	1.00	1.00
	Yes	1.12 (0.79–1.60)	1.07 (0.75–1.53)	0.81 (0.58–1.14)	1.08 (0.69–1.68)	1.03 (0.64–1.67)	0.76 (0.47–1.22)
Binge drinking^#^ (past 30 days)	No	1.00	1.00	1.00	1.00	1.00	1.00
	Yes	1.74 (1.15–2.65)^b^	1.62 (1.05–2.52)^a^	1.43 (0.90–2.28)	1.15 (0.78–1.70)	1.10 (0.72–1.69)	0.79 (0.44–1.41)
**Sexual behaviour**							
Lifetime sex	No	1.00	1.00	1.00	1.00	1.00	1.00
	Yes	1.79 (1.27–2.54)^a^	1.62 (1.16–2.26)^b^	1.28 (0.91–1.80)	1.60 (0.98–2.61)	1.56 (1.01–2.42)^a^	1.36 (0.80–2.33)
Last sex alcohol or drug use^$^	No	1.00	1.00	1.00	1.00	1.00	1.00
	Yes	1.19 (0.36–3.91)	1.10 (0.27–4.41)	0.78 (0.15–4.10)	2.10 (1.20–3.69)^a^	2.18 (1.32–3.58)^b^	1.89 (0.83–4.34)
Last sex non-condom use^$,*^	No	1.00	1.00	1.00	1.00	1.00	1.00
	Yes	1.58 (0.70–3.59)	1.38 (0.54–3.54)	1.58 (0.56–4.44)	0.96 (0.37–2.52)	1.05 (0.40–2.79)	0.79 (0.28–2.20)
Got someone pregnant/been pregnant^*^	No	1.00	1.00	1.00	1.00	1.00	1.00
	Yes	2.22 (1.34–3.69)^b^	1.88 (1.15–3.09)^a^	1.69 (1.12–2.54)^a^	3.00 (1.90–4.72)^c^	2.99 (1.85–4.82)^c^	1.65 (0.81–3.36)
**Violence** (past year)							
Started a fistfight or shoving match	No	1.00	1.00	1.00	1.00	1.00	1.00
	Yes	0.92 (0.64–1.33)	0.88 (0.58–1.36)	0.76 (0.50–1.16)	0.82 (0.61–1.11)	0.86 (0.63–1.16)	0.67 (0.41–1.11)
Hurt someone badly in a physical fight	No	1.00	1.00	1.00	1.00	1.00	1.00
	Yes	0.99 (0.46–2.14)	1.04 (0.46–2.35)	0.72 (0.30–1.75)	1.54 (0.96–2.47)	1.78 (1.08–2.94)^a^	1.05 (0.51–2.19)
Carried a blade, knife, or gun in school	No	1.00	1.00	1.00	1.00	1.00	1.00
	Yes	1.04 (0.54–1.99)	1.08 (0.56–2.07)	0.90 (0.42–1.92)	1.64 (1.23–2.18)^b^	1.75 (1.29–2.39)^c^	1.09 (0.62–1.90)

^¤^In response to the statement ‘I felt lonely (in the past 30 days)’, ‘Not true’ and ‘Somewhat true’ were used as the reference category (No) while ‘Certainly true’ was taken as signifying feeling lonely (Yes).

^†^Adjusted for age, family structure, and parental education.

^ǂ^Adjusted for age, family structure, parental education, and depressive symptoms.

^§^Illicit drug use includes huffing to get high (glue, aerosols), amphetamines, ecstasy or dust, LSD (Acid) or PCP.

^¶^Alcohol consumption was based on the consumption of either beer, wine or hard liquor.

^#^Binge drinking referred to drinking at least five drinks of alcohol in a row on at least one day.

^$^Restricted to those who ever had sex.

^*^Age categories of 13 and 14 years among girls were collapsed for adjustment due to the small number of events in the youngest category.

^a^P < 0.05, ^b^P < 0.01, ^c^P < 0.001.

In the univariate analysis, lonely U.S. girls had significantly higher odds for eight of the 12 health risk behaviours (Table 3). In the final model (Model 3) however, over half of these associations had been significantly attenuated (when controlling for depressive symptoms) so that only different types of substance use continued to be linked to loneliness. Lonely U.S. girls were thus more likely to have used marijuana (OR, 1.79; CI, 1.26–2.55), consumed alcohol (OR, 1.80; CI, 1.18–2.75) and engaged in binge drinking (OR, 2.40; CI, 1.56–3.70). American boys who were lonely were more likely to binge drink, consume alcohol and use other illicit drugs in the univariate analysis (Table 3). In Model 3, only this latter association with illicit drug use remained statistically significant (OR, 3.09: CI, 1.41–6.77).

**Table 3:** Univariate and multivariate analyses on the association between loneliness and health risk behaviours among U.S. adolescents

		**Girls**			**Boys**		
		**Model 1**	**Model 2**	**Model 3**	**Model 1**	**Model 2**	**Model 3**
**Health risk behaviours (outcome)**	**I felt lonely**^**¤**^	**OR (95% CI)**	**adj. OR (95% CI)**^**†**^	**adj. OR (95% CI)**^**ǂ**^	**OR (95% CI)**	**adj. OR (95% CI)**^**†**^	**adj. OR (95% CI)**^**ǂ**^
**Substance use**							
Smoking (past 30 days)	No	1.00	1.00	1.00	1.00	1.00	1.00
	Yes	2.56 (1.49–4.38)^b^	2.56 (1.47–4.44)^b^	1.86 (0.88–3.94)	1.11 (0.34–3.61)	0.96 (0.29–3.22)	0.72 (0.17–2.97)
Lifetime marijuana use	No	1.00	1.00	1.00	1.00	1.00	1.00
	Yes	2.11 (1.48–3.02)^c^	2.08 (1.41–3.06)^c^	1.79 (1.26–2.55)^b^	1.96 (0.97–3.96)	1.68 (0.83–3.42)	1.17 (0.48–2.87)
Lifetime other illicit drug use^§^	No	1.00	1.00	1.00	1.00	1.00	1.00
	Yes	3.00 (1.66–5.41)^c^	3.03 (1.69–5.42)^c^	1.72 (0.97–3.07)	4.44 (2.27–8.69)^c^	4.30 (2.37–7.82)^c^	3.09 (1.41–6.77)^b^
Alcohol consumption^¶^ (past 30 days)	No	1.00	1.00	1.00	1.00	1.00	1.00
	Yes	2.71 (1.83–4.01)^c^	2.68 (1.75–4.09)^c^	1.80 (1.18–2.75)^b^	2.08 (1.20–3.59)^b^	1.83 (1.03–3.25)^a^	0.87 (0.40–1.87)
Binge drinking^#^ (past 30 days)	No	1.00	1.00	1.00	1.00	1.00	1.00
	Yes	3.60 (2.21–5.85)^c^	3.57 (2.14–5.96)^c^	2.40 (1.56–3.70)^c^	2.60 (1.16–5.83)^a^	2.22 (1.01–4.87)^a^	1.05 (0.36–3.07)
**Sexual behaviour**							
Lifetime sex	No	1.00	1.00	1.00	1.00	1.00	1.00
	Yes	1.79 (1.14–2.82)^a^	1.77 (1.11–2.84)^a^	1.29 (0.76–2.19)	1.41 (0.89–2.24)	1.25 (0.75–2.11)	1.13 (0.61–2.10)
Last sex alcohol or drug use^$^	No	1.00	1.00	1.00	1.00	1.00	1.00
	Yes	6.24 (1.62–23.99)^b^	6.22 (1.54–25.08)^a^	4.21 (0.80–22.22)	2.44 (0.53–11.22)	1.97 (0.42–9.20)	2.49 (0.31–20.21)
Last sex non-condom use^$,*^	No	1.00	1.00	1.00	1.00	1.00	1.00
	Yes	2.87 (0.90–9.21)	2.67 (0.83–8.56)	2.74 (0.80–9.41)	0.92 (0.36–2.38)	1.00 (0.38–2.59)	0.77 (0.24–2.48)
Got someone pregnant/been pregnant^*^	No	1.00	1.00	1.00	1.00	1.00	1.00
	Yes	2.96 (0.66–13.37)	3.02 (0.69–13.25)	1.57 (0.31–8.05)	1.87 (0.72–4.82)	1.74 (0.70–4.37)	0.66 (0.18–2.38)
**Violence** (past year)							
Started a fistfight or shoving match	No	1.00	1.00	1.00	1.00	1.00	1.00
	Yes	1.26 (0.90–1.76)	1.23 (0.89–1.71)	0.84 (0.59–1.20)	1.36 (0.84–2.19)	1.30 (0.81–2.08)	0.89 (0.50–1.57)
Hurt someone badly in a physical fight	No	1.00	1.00	1.00	1.00	1.00	1.00
	Yes	1.76 (1.26–2.45)^b^	1.77 (1.27–2.49)^b^	1.24 (0.76–2.01)	1.92 (0.85–4.35)	1.69 (0.72–3.98)	1.25 (0.48–3.25)
Carried a blade, knife, or gun in school	No	1.00	1.00	1.00	1.00	1.00	1.00
	Yes	1.65 (0.93–2.91)	1.59 (0.89–2.85)	0.99 (0.50–1.95)	1.85 (0.98–3.51)	1.68 (0.78–3.63)	0.81 (0.31–2.07)

^¤^In response to the statement ‘I felt lonely (in the past 30 days)’, ‘Not true’ and ‘Somewhat true’ were used as the reference category (No) while ‘Certainly true’ was taken as signifying feeling lonely (Yes).

^†^Adjusted for age, family structure, and parental education.

^ǂ^Adjusted for age, family structure, parental education, and depressive symptoms.

^§^Illicit drug use includes huffing to get high (glue, aerosols), amphetamines, ecstasy or dust, LSD (Acid) or PCP.

^¶^Alcohol consumption was based on the consumption of either beer, wine or hard liquor.

^#^Binge drinking referred to drinking at least five drinks of alcohol in a row on at least one day.

^$^Restricted to those who ever had sex.

^*^Age categories of 14 and 15 years among girls were collapsed for adjustment due to the small number of events in the oldest category.

^a^P < 0.05, ^b^P < 0.01, ^c^P < 0.001.

## Discussion

This study has shown that loneliness is commonplace among adolescents in both Russia and the U.S., and that feeling lonely is associated with adolescent health risk behaviours, in particular, substance use. There is also some indication that other factors may be important in the association between adolescent loneliness and health risk behaviour. Specifically, in several instances when the depressive symptoms variable was added to the statistical analysis, the strength of the relation between loneliness and the health risk behaviours was attenuated so that previously statistically significant associations (e.g. between loneliness and smoking in Russian and U.S female adolescents) became non-significant.

As yet, there has been little cross-cultural research on the phenomenon of adolescent loneliness and how its occurrence varies between countries. Even where such data are available, making comparisons is complicated by the different ways in which loneliness has been defined and measured across studies. In the present study, 6.7% (boys) to 14.7% (girls) of U.S. students reported that they felt lonely while the corresponding figures among Russian students were 8.9% (boys) and 14.4% (girls). These figures accord with the findings from a recent multi-country study where (excepting Chile) the prevalence of often feeling lonely was similar among boys and girls in the Philippines, Namibia, and China, with the figures for these countries ranging from 6.6% to 13.1% [16].

As mentioned in the introduction, although some studies have suggested that there may be a link between loneliness and the occurrence of aggressive behaviour [44] as well as between adolescent rejection and aggression [5], until now, there has been comparatively little systematic research on the relationship between adolescent loneliness and different forms of aggression and violent behaviour. In the current study, there was no relation in the multivariate analysis between feeling lonely and any form of violent behaviour for either boys or girls in Russia or the United States. This finding accords with that from an earlier study in the capital of Thailand, Bangkok, where there was no association found between loneliness and either fighting or weapon carrying [29]. It also corresponds with the result from a recent study which similarly found no association between loneliness and aggressive behaviour among U.S. adolescents. There it was suggested that aggressive adolescents may group together with other aggressive children and in that way, avoid feelings of loneliness [45].

It should be noted however, that in the model adjusted for demographic characteristics, loneliness was linked to higher odds for both weapon carrying (Russian boys) and hurting someone badly in a fight (Russian boys, U.S. girls) but that these relations were attenuated when controlling for depressive symptoms. This together with other results from the analysis suggests that adolescent depression may be an important mediator of the pathway between adolescent loneliness and some health risk behaviours and that it should therefore be considered when examining potential associations between these variables. It also highlights the importance of longitudinal studies to better elucidate the relation between adolescent loneliness and health risk behaviours and how other factors, such as depressive symptoms, affect this relation. As regards violent behaviour, this would seem especially necessary given that a previous study from the U.S. has shown that depressive symptoms may be linked to an increased risk for violent behaviour among adolescents [46].

As regards sexual risk behaviours, although several significant associations were observed in the univariate analysis, in the fully-adjusted analysis loneliness was only associated with having been pregnant among Russian girls. Engaging in sexual activity may be one way of trying to negate the effects of loneliness [47] by connecting physically with others, with the obvious risk this carries for future pregnancy. Given that our study was limited to adolescents aged 13–15 years old and that there are exceptionally high rates of adolescent abortion in Russia [48], it is possible that many of the girls who became pregnant may not have given birth. However, for those adolescents who did, it is possible that pregnancy itself might have been a precursor of loneliness by necessitating continued close reliance on parents while undermining the possibility of greater peer involvement. This might have resulted in both poorer self perceptions and greater feelings of social isolation and loneliness [49]. If this is one pathway that links adolescent pregnancy and loneliness it might be especially important in Russia as research has indicated that adolescent boys and girls spend a greater percentage of their free time with peers than young people in many other counties [50].

Feeling lonely was most strongly linked to adolescent substance use in the current study. Lonely boys and girls in Russia and the U.S. all had higher odds for engaging in at least one type of substance use risk behaviour. This finding accords with those from several previous studies [14,16,28]. As mentioned in the introduction, substance use might be a means of self-medication and of alleviating the negative feelings that emanate from being lonely [16]. Alternatively, these behaviours might be undertaken in an attempt to reach out and gain peer approval [51]. If this is happening it is possible that Russian and U.S. adolescents may have been using the substance(s) that they perceived as being best or most easily suited to this goal. The prevalence of past 30-day cigarette use among Russian adolescent males for example, is one of the highest in Europe [52]. In such circumstances, it might be easier for lonely Russian boys to connect with others by using this substance. Some support for this speculation comes from a recent U.S. study which showed that the link between loneliness and smoking among late adolescents was strongest in a region of the country (the Midwest) where the prevalence of smoking was higher [53]. Given the marked differences in the association between loneliness and different forms of substance use among adolescent boys and girls within and between countries, this highlights the importance of qualitative research being undertaken in the future in order to elucidate the precise mechanisms underlying the association between loneliness and variations in adolescent substance use.

There are several potential limitations to this study. We used a single item question to measure loneliness. This might have been problematic as loneliness has been described as a complex phenomenon involving feelings of deprivation, differing emotional components and a temporal perspective [54]. As such, it has been claimed that multiple item scales are preferable when examining this phenomenon [55]. It has also been argued however, that for single item questions, categories at both ends of the scale (i.e. not lonely/severely lonely) are ‘broadly robust’ and produce prevalence figures comparable with those from multi-item scales [56]. In addition, we had to rely on adolescent self-reports of particular health risk behaviours with no way of confirming their accuracy with the potential for reporting bias that this carries. A previous study that examined adolescent smoking in Russian Karelia suggested for example, that girls might have under-reported their own smoking as it was (still) less culturally acceptable for females to smoke [57]. The data were also drawn from single study sites in both countries and therefore the results might not be representative countrywide. Moreover, for some of the variables there were a moderate number of missing cases that could have biased the results of this study. For example, data on sexual intercourse were missing from 13.9% and 16.6% of Russian and American boys respectively. In addition, we were not able to control for certain variables that might affect the relation between loneliness and health risk behaviour. Some evidence suggests for example, that personality factors such as low self-esteem may be linked to both loneliness and the onset of certain health risk behaviours [4,58]. Lastly, because of the cross-sectional nature of these data we had no way of determining the temporal ordering of the associations we observed.

## Conclusion

This study has shown that loneliness is associated with health risk behaviours (primarily substance use) among adolescent boys and girls in Russia and the United States. It has also highlighted that other factors such as adolescent mental health may be important when assessing this relation and will need to be considered in future studies. Longitudinal research using both quantitative and qualitative methodology is now needed to better understand adolescent loneliness and how it is linked to health risk behaviours in order to facilitate the development of interventions that address loneliness and its potentially harmful effects on adolescent well-being.

## Competing interests

The authors declare that they have no competing interests.

## Authors’ contributions

AS conceived the study idea, interpreted the data and wrote the main body of the text. AK did the data analysis, interpreted the data and commented on the manuscript for intellectual content. RK, MS-S and VR organised and undertook the survey, and contributed to the drafting of the manuscript. All authors read and approved the final manuscript.

## Pre-publication history

The pre-publication history for this paper can be accessed here:

http://www.biomedcentral.com/1471-2458/14/366/prepub
